# Generative AI use and social disparities in pediatric rehabilitation: a cross-sectional study of ChatGPT use among parents of children with speech and language disorders in Türkiye

**DOI:** 10.3389/fpubh.2026.1817290

**Published:** 2026-06-08

**Authors:** Agit Şimşek, Zehra Güneş

**Affiliations:** 1 Department of Speech and Language Therapy, Inonu University, Malatya, Türkiye; 2 Department of Audiology, Inonu University, Malatya, Türkiye

**Keywords:** ChatGPT, digital divide, generative artificial intelligence, pediatric rehabilitation, speech and language disorders

## Abstract

**Background:**

Generative artificial intelligence (AI) tools such as ChatGPT are increasingly used for health information seeking. However, little is known about how the adoption of generative AI is socially patterned, particularly in pediatric rehabilitation contexts. This study aimed to examine the prevalence and sociodemographic determinants of ChatGPT use among parents of children with speech and language disorders (SLDs) in Türkiye and to explore its association with engagement in formal speech and language therapy services.

**Methods:**

This cross-sectional study included 204 parents of children with SLDs recruited through online parent networks between October and November 2025. Data were collected using a structured questionnaire assessing sociodemographic characteristics, therapy attendance frequency, and ChatGPT use for SLD-related information. Bivariate associations were analyzed using chi-square tests, and multivariable logistic regression was conducted to identify independent predictors of ChatGPT use.

**Results:**

Overall, 28.4% of participants reported using ChatGPT to seek information related to their child’s speech or language disorder. ChatGPT use was significantly associated with gender, age group, and educational level. In multivariable analyses, male gender (aOR = 3.09; 95% CI: 1.36–7.02) and postgraduate education (aOR = 5.20; 95% CI: 1.56–17.27) were independently associated with higher odds of ChatGPT use, while undergraduate education did not reach statistical significance (aOR = 1.55; 95% CI: 0.70–3.42; *p* = 0.277). Attending therapy several times per year (aOR = 4.01; 95% CI: 1.08–14.92) and monthly or weekly (aOR = 2.75; 95% CI: 1.01–7.48) were both associated with higher odds of ChatGPT use relative to never attending therapy.

**Conclusion:**

Generative AI use among parents of children with SLDs appears relatively common but socially stratified. Educational attainment, gender, and engagement with formal therapy services significantly influence AI adoption. Without equity-oriented strategies and digital health literacy initiatives, the diffusion of generative AI tools may reinforce existing informational disparities in pediatric rehabilitation.

## Introduction

The rapid diffusion of generative artificial intelligence (AI) tools, particularly large language models (LLMs) such as ChatGPT, has transformed the global health information ecosystem (1, 2). In parallel, advances in digital health infrastructures and data-driven technologies have expanded the role of intelligent systems in healthcare delivery (3). At the same time, growing evidence highlights the complexity of mental health conditions, which are shaped by multidimensional biological and psychosocial mechanisms, underscoring the need for integrative approaches in healthcare research (4). Recent developments in intelligent health monitoring and AI-enabled biomedical systems further demonstrate how data-driven models are increasingly integrated into clinical decision-making and patient support environments (29), expanding the functional scope of digital health ecosystems beyond information retrieval (5).

Among generative AI tools, ChatGPT represents one of the most widely accessible and rapidly adopted large language models globally. Its conversational, user-friendly interface and ability to generate context-specific responses have made it a prominent tool for health information seeking among the general public. As such, ChatGPT provides a relevant and practical proxy for examining real-world patterns of generative AI use in health-related contexts. Unlike traditional web searches, these systems generate conversational, context-responsive responses that appear personalized and accessible, increasingly functioning as informal sources of health-related guidance for patients and caregivers. However, the broader public health implications of this technological shift remain insufficiently understood. Recent evidence underscores the accelerating integration of generative AI into everyday health information practices. A 2026 multinational study across four European countries found that generative AI use for health information is currently driven by early adopters—those who find it useful, easy to integrate, and possess the necessary digital skills—with cross-national consistency suggesting a shared adoption pattern (6). In the pediatric context specifically, a dual-evaluation study involving clinical experts and caregivers identified a significant trust-accuracy gap: while medical experts highlighted inaccuracies in over a quarter of AI-generated responses, the majority of parents expressed high trust and perceived the information as helpful (7). Furthermore, a cross-sectional survey conducted in early 2025 among 185 family members of children receiving hospital care found considerable awareness and use of large language models—including ChatGPT—for pediatric health-related queries (8). Collectively, these developments highlight the urgency of examining who uses generative AI for health information, under what conditions, and with what implications for equitable access to reliable guidance. Across these studies, a consistent pattern emerges: generative AI use for health information is not uniform but is shaped by users’ digital skills, trust dispositions, and contextual factors. However, none of these studies have examined how AI adoption intersects with engagement in formal rehabilitation services—a gap this study directly addresses (6). Beyond conversational interfaces, recent studies highlight the growing role of AI-driven systems in personalized health monitoring, predictive analytics, and adaptive user interaction, suggesting that generative AI tools are part of a broader ecosystem of intelligent healthcare technologies (9).

Digital health technologies have long been associated with disparities in access and use, commonly conceptualized as the “digital divide.” While early discussions focused primarily on access to internet infrastructure, contemporary scholarship emphasizes a second-level digital divide characterized by differences in digital literacy, confidence, and the ability to critically evaluate digital information (10). Van Deursen and Van Dijk (11) further demonstrated that second-level digital inequalities are strongly mediated by socioeconomic status and formal education, underscoring the structural roots of differential digital engagement. This second-level divide is particularly relevant in health contexts, where disparities in digital competence translate into unequal capacity to locate, interpret, and critically evaluate health-related information online. The expansion of online health content has also raised concerns regarding misinformation and its potential effects on healthcare utilization (12, 13). Generative AI may represent a new phase of this divide, as effective engagement requires not only technological access but also the capacity to interpret, contextualize, and verify AI-generated outputs (1). Beyond traditional digital health tools, generative AI introduces distinct interactional demands that may uniquely shape health information inequities. Unlike static web-based information, large language models require users to actively formulate prompts, interpret probabilistic outputs, and critically evaluate potentially uncertain or hallucinated responses. This shifts part of the informational burden from system design to user capability. As a result, individuals with higher cognitive, educational, and digital competencies may disproportionately benefit from these tools, while others may face increased risks of misinterpretation or misinformation. In this sense, generative AI may not only reflect but actively amplify second-level digital inequalities.

In pediatric rehabilitation, equitable access to professional services remains a persistent public health concern. Speech and language disorders (SLDs) affect children’s cognitive, academic, and psychosocial development and require early identification and consistent therapeutic intervention to prevent long-term adverse outcomes (14, 15). Early and consistent access to speech-language therapy has been shown to substantially improve long-term language outcomes; however, service uptake remains inequitably distributed across socioeconomic and geographic groups in many countries (16). In Türkiye specifically, geographic disparities in access to speech-language pathology services have been documented, with families in rural and underserved areas facing particular challenges in reaching qualified clinicians (17).

Parents play a central role in recognizing developmental concerns, seeking appropriate care, and navigating healthcare systems (15). If AI-based tools are disproportionately adopted by certain demographic groups—or used as substitutes in contexts of limited service access—they may reinforce or reshape existing inequities in rehabilitation engagement.

The Technology Acceptance Model (TAM), originally proposed by Davis (18), provides a foundational framework for understanding individual adoption of information technologies. TAM posits that two primary cognitive constructs drive behavioral intention to use a technology: Perceived Usefulness (PU), defined as the degree to which an individual believes that using a particular system will enhance their performance, and Perceived Ease of Use (PEOU), defined as the degree to which a person believes that using a system will be free of effort. According to TAM, PEOU directly influences PU, and both constructs jointly shape behavioral intention, which in turn predicts actual technology use. The model has been widely validated across health technology contexts, demonstrating robust explanatory power for predicting patient and caregiver engagement with digital health tools. TAM has been applied across a broad range of digital health contexts, including caregiver engagement with health information tools and adoption of AI-based health applications, consistently demonstrating that perceived ease of use and perceived usefulness are central drivers of adoption behavior (19, 20).

When applied to generative AI tools such as ChatGPT, the TAM framework requires conceptual extension beyond its original scope. Unlike conventional software applications, large language models present a distinctively conversational and context-adaptive interface that may lower perceived barriers to use for some groups while simultaneously introducing new forms of complexity for others. In this study, PU is operationalized as the degree to which parents perceive ChatGPT as a useful source of information for understanding and managing their child’s speech and language disorder. PEOU refers to the degree to which parents perceive interacting with ChatGPT as requiring minimal cognitive effort. Critically, both constructs are hypothesized to be moderated by sociodemographic factors—specifically educational attainment, age, and gender—which shape the capacity to engage with and critically appraise AI-generated health content. These external variables are treated as antecedents to TAM constructs in the present model, reflecting the second-level digital divide perspective: differential ability to benefit from technology, not merely access to it. Furthermore, therapy attendance frequency is incorporated as a structural context variable, capturing whether formal professional engagement reduces reliance on informal AI-based information sources—a dynamic that extends beyond standard TAM formulations.

Despite growing interest in AI in healthcare (1, 2), empirical evidence regarding parental use of generative AI in pediatric neurodevelopmental conditions remains scarce, particularly in middle-income countries. It remains unclear whether generative AI functions primarily as a bridge that enhances health engagement or as a parallel—and potentially substitutive—information pathway in the absence of professional care.

Despite the growing integration of generative AI into everyday health information seeking, it remains unknown to what extent parents of children with speech and language disorders use ChatGPT for disorder-related information, whether this use is socially stratified, and whether it relates to engagement with formal rehabilitation services. This represents a critical gap, as unexamined and inequitably distributed AI use in pediatric rehabilitation contexts may silently reinforce existing health disparities without being detected by current clinical or public health monitoring systems (7, 8).

Therefore, this cross-sectional study aimed to examine the prevalence and determinants of ChatGPT use among parents of children with speech and language disorders (SLDs) in Türkiye and to investigate its association with engagement in formal speech-language therapy services. Drawing on the Technology Acceptance Model and digital divide frameworks, we hypothesized that younger age and higher educational attainment would be positively associated with ChatGPT use. We further explored whether parents whose children did not receive regular therapy would be more likely to use ChatGPT, reflecting a potential compensatory dynamic in contexts of limited clinical engagement. Here, “compensatory” refers to the use of informal digital tools—such as AI—as a substitute when access to formal professional services is limited or infrequent.

## Methods

### Study design and setting

This cross-sectional study was conducted to examine the prevalence and determinants of generative artificial intelligence (AI) use among parents of children with speech and language disorders (SLDs) in Türkiye. Data were collected between October and November 2025 using an online survey platform.

The study was designed and reported in accordance with the Strengthening the Reporting of Observational Studies in Epidemiology (STROBE) guidelines for cross-sectional research.

### Participants and recruitment

Participants were parents residing in Türkiye who had at least one child diagnosed with a speech and language disorder. Eligibility criteria included:

- being a parent or primary caregiver of a child with a reported SLD diagnosis,
- being 18 years of age or older,
- having sufficient Turkish language proficiency to complete the questionnaire,
- providing informed consent.

The age threshold of 18 years was selected to ensure that all participants were legally recognized adults capable of providing independent informed consent, consistent with standard ethical practice in survey-based research involving caregivers. While younger individuals may serve as primary caregivers in some contexts, restricting participation to adults aged 18 and above ensured that consent was obtained without the need for parental or guardian authorisation, thereby simplifying the ethical framework and strengthening the integrity of the consent process.

Participants were recruited through online parent networks, social media groups related to speech and language disorders, and professional communication channels. This recruitment strategy was selected to reflect real-world digital information environments in which parents commonly seek health-related information.

Because recruitment occurred through online platforms, the sample is likely to overrepresent parents with higher levels of digital engagement and literacy. This has an important methodological implication: ChatGPT use in this sample may be systematically overestimated relative to the broader population of parents of children with SLDs, including those with limited internet access. Researchers and readers should therefore exercise caution when generalizing these findings beyond digitally active populations.

Incomplete questionnaires (defined as missing more than 50% of responses) and internally inconsistent responses were excluded. Missing data were minimal (<5%) and were handled using complete-case analysis.

A total of 204 parents met inclusion criteria and were included in the final analyses.

To ensure adequate statistical power for multivariable logistic regression analyses, a minimum ratio of 10 outcome events per predictor variable was targeted. Given the number of predictors included in the final model, the achieved sample size was considered sufficient to ensure statistical stability. Additionally, a post-hoc power analysis conducted using G*Power 3.1 indicated that the sample size (*N* = 204) provided over 90% power to detect medium effect sizes (*α* = 0.05) in primary association analyses.

### Measures

#### Sociodemographic variables

Participants reported age (categorized as 18–24, 25–34, 35–44, and ≥45 years), gender (female/male), educational level, and monthly household income.

For multivariable analyses, educational level was retained as a three-category variable: high school or below, undergraduate, and postgraduate. This categorization preserves the distinct educational strata observed in the sample and allows for the detection of differential associations across education levels in the regression model.

Household income was dichotomized according to national median income thresholds.

#### Questionnaire development

The questionnaire used in this study was developed specifically for the present research and has not been previously published. An initial pool of 40 items was generated based on the study objectives and relevant literature on children’s language and speech development. Item generation was specifically informed by previously published frameworks and instruments used in digital health information-seeking research, including studies on parental health information behavior, technology acceptance in healthcare contexts, and AI literacy assessment tools. Specifically, items related to parental health information behavior were informed by Baumann et al. (10); items assessing technology acceptance were guided by the original TAM scales of Davis (18); and items pertaining to AI use for health information were developed with reference to Ayo-Ajibola et al. (19) and Matthes et al. (6). The preliminary version of the questionnaire was independently reviewed by three experts in the fields of speech and language therapy and audiology to assess content relevance, clarity, and appropriateness.

Following expert feedback, items were revised, merged, or removed, resulting in a final 22-item structured questionnaire. The revised version was subsequently pilot-tested in a small group of parents to evaluate comprehensibility and response feasibility, after which no further modifications were required.

The final questionnaire addresses multiple domains related to children’s language and speech development and focuses on parental perspectives, including sociodemographic characteristics, sources of information, experiences with speech and language concerns, and the use of artificial intelligence–based tools such as ChatGPT.

An English-language version of the questionnaire used in the present study is provided as Supplementary File 1 and is cited in the main manuscript.

#### Child-related clinical variables

Parents reported their child’s speech and language diagnosis (multiple responses permitted) and therapy attendance frequency (never attended; infrequent—several times per year/once per month; weekly attendance).

Therapy attendance frequency was included as a key explanatory variable to examine associations between AI use and engagement with formal rehabilitation services.

#### Primary outcome variable

The primary outcome was defined as self-reported use of ChatGPT specifically for speech and language disorder–related information. Participants were asked:

“Have you ever used ChatGPT to seek information or advice specifically regarding your child’s speech or language condition or therapy process?”

Responses were coded as a binary variable (Yes/No). General or unrelated use of ChatGPT was not considered part of the primary outcome.

### Perceptions of ChatGPT (TAM-based measures)

TAM-relevant perceptions were assessed using items from the structured questionnaire that capture constructs conceptually aligned with Perceived Usefulness (PU) and Perceived Ease of Use (PEOU), without employing a formally validated TAM instrument. PU was operationalized through items assessing the perceived advantages of ChatGPT for health information seeking (e.g., quick access to information, early awareness and guidance, personalized suggestions). PEOU was operationalized through items assessing perceived practical accessibility and ease of interaction (e.g., 24/7 availability, low cost, ability to provide practical answers to non-complex questions). Participants indicated which advantages and accessibility features they associated with ChatGPT (binary endorsement format), and mean endorsement proportions were computed for each construct.

Internal consistency for these items in the present sample was acceptable (Cronbach’s alpha = 0.78). Given that the items were developed specifically for this study rather than drawn from a standardized TAM instrument, findings related to PU and PEOU should be interpreted as indicative rather than as formal psychometric measures.

### Statistical analysis

All statistical analyses were performed using IBM SPSS Statistics version 25 (IBM Corp., Armonk, NY, United States).

Descriptive statistics were reported as frequencies, percentages, means, and standard deviations as appropriate.

Bivariate associations between ChatGPT use and categorical variables were examined using chi-square (χ^2^) tests. Yates’ continuity correction was applied for 2 × 2 comparisons where appropriate.

Multivariable binary logistic regression analysis was conducted to identify independent predictors of ChatGPT use. The dependent variable was ChatGPT use for SLD-related information (Yes/No). Independent variables included gender, age group, educational level (high school or below; undergraduate; postgraduate), household income, and therapy attendance frequency.

Independent variables were selected based on theoretical relevance, prior literature on digital health behavior, and the Technology Acceptance Model framework. All variables were entered simultaneously into the regression model to adjust for potential confounding effects.

Adjusted odds ratios (aORs) with 95% confidence intervals (CIs) were reported. Multicollinearity was assessed using variance inflation factors (VIF), with values below 2.0 considered acceptable. Model fit was evaluated using the Hosmer–Lemeshow goodness-of-fit test. All model assumptions were assessed and met.

A two-tailed *p*-value of < 0.05 was considered statistically significant.

### Ethical considerations

This study was approved by the Ethics Committee of Inonu University (Non-Interventional Clinical Research Ethics Committee) (Session 20, Decision No: 22, 03 October 2025). All methods were carried out in accordance with the 2013 Declaration of Helsinki and relevant institutional guidelines. All participants were informed about the purpose of the study, assured of confidentiality, and provided electronic written informed consent prior to participation. Participation was voluntary, and respondents could withdraw at any time without consequence.

No personally identifiable information was collected. IP filtering was used solely to prevent duplicate responses and was not stored or linked to survey data.

## Results

### Participant characteristics

A total of 204 parents of children with speech and language disorders (SLDs) were included in the analysis. Sociodemographic and clinical characteristics are presented in Table 1.

**Table 1:** Sociodemographic and clinical characteristics of the participants (*N* = 204).

Characteristic	Category	*N*	%
Gender	Female	148	72.5
	Male	56	27.5
Age group	18–24	15	7.4
	25–34	61	29.9
	35–44	84	41.2
	≥45	44	21.6
Educational level	High school or below	101	49.5
	Undergraduate	82	40.2
	Postgraduate	21	10.3
Monthly income	≤44,000 TL	115	56.4
	>44,000 TL	89	43.6
Therapy attendance	Never	42	20.6
	Infrequent	24	11.8
	Weekly	138	67.6

Of the participants, 72.5% (*n* = 148) were female and 27.5% (*n* = 56) were male. The largest age group was 35–44 years (41.2%), followed by 25–34 years (29.9%), ≥45 years (21.6%), and 18–24 years (7.4%).

Regarding educational level, 49.5% (*n* = 101) had completed high school or below, 40.2% (*n* = 82) held an undergraduate degree, and 10.3% (*n* = 21) had postgraduate education. More than half of the participants (55.7%) reported a monthly household income of ≤44,000 TL.

Children’s diagnoses (multiple responses permitted) included hearing loss (46.1%), developmental language disorder (31.4%), articulation disorder (12.3%), and stuttering (11.3%).

In terms of therapy engagement, 67.6% of children attended speech and language therapy on a monthly or weekly basis, 11.8% attended infrequently (several times per year), and 20.6% had never attended therapy.

### Prevalence of ChatGPT use

Overall, 58 participants (28.4%) reported using ChatGPT for speech and language disorder–related information, while 146 (71.6%) reported no use.

#### TAM-based perceptions of ChatGPT

TAM-relevant perceptions were examined among all 204 participants regardless of ChatGPT use status, using questionnaire items conceptually aligned with Perceived Usefulness (PU) and Perceived Ease of Use (PEOU). Regarding PEOU, ChatGPT users endorsed a substantially higher proportion of practical accessibility items (e.g., 24/7 availability, low cost, practical answers) compared to non-users, and this difference was statistically significant (Mann–Whitney U = 6273.5, *p* < 0.001), indicating that users perceived the tool as considerably more practically accessible. Regarding PU, endorsement of perceived usefulness items (e.g., early awareness, personalized suggestions, home-based support) did not significantly differ between users and non-users (Mann–Whitney U = 4378.5, *p* = 0.637). These patterns offer partial support for TAM’s theoretical propositions: while PEOU differentiated adopters from non-adopters as predicted, PU did not reach significance in this sample, possibly reflecting the binary operationalization of these constructs rather than an absence of usefulness perceptions among users. Given that these items were not drawn from a standardized TAM instrument, findings should be interpreted as indicative rather than as formal psychometric measures.

### Bivariate associations

**Figure 1:**
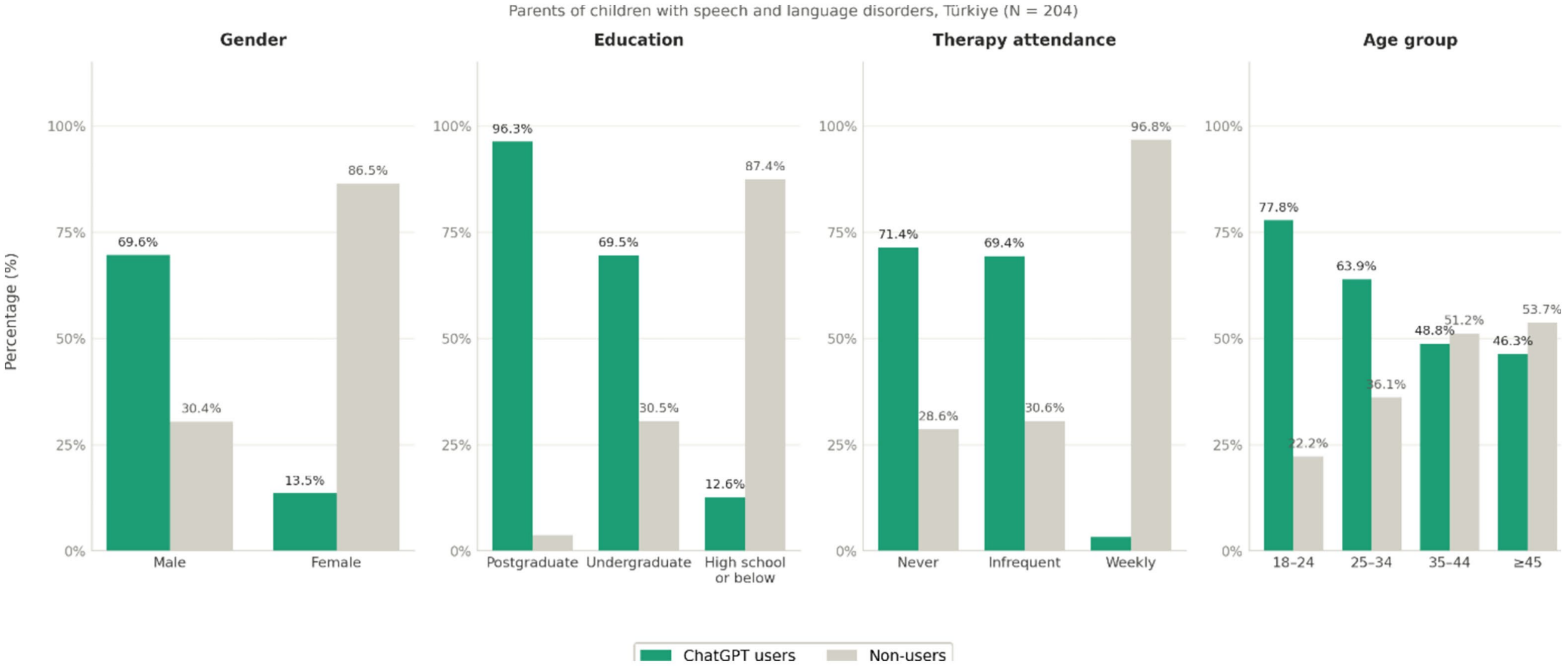
ChatGPT use rates (%) among parents of children with speech and language disorders by gender, educational level, therapy attendance frequency, and age group (*N* = 204). Green bars = ChatGPT users; grey bars = non-users.

Associations between ChatGPT use and demographic and clinical factors are presented in Table 2 and Figure 1.

**Table 2:** Distribution of independent variables by ChatGPT use.

Variable/category	Non-users *n* (%) *n* = 146	Users *n* (%) *n* = 58	chi2	*P*
Age group			**9.217**	0.027*
18–24	6 (40.0%)	9 (60.0%)		
25–34	42 (68.9%)	19 (31.1%)		
35–44	65 (77.4%)	19 (22.6%)		
45 and above	33 (75.0%)	11 (25.0%)		
Gender			**8.902**	0.003*
Female	115 (77.7%)	33 (22.3%)		
Male	31 (55.4%)	25 (44.6%)		
Educational level			**9.249**	0.006*
High school or below	80 (79.2%)	21 (20.8%)		
Undergraduate	56 (68.3%)	26 (31.7%)		
Postgraduate	9 (45.0%)	11 (55.0%)		
Income level			**5.078**	**0.079**
0–22,000 TL	33 (84.6%)	6 (15.4%)		
22,000–44,000 TL	47 (64.4%)	26 (35.6%)		
Above 44,000 TL	63 (70.8%)	26 (29.2%)		
SLT attendance frequency			**5.034**	**0.081**
Never attended	35 (83.3%)	7 (16.7%)		
Several times a year	14 (58.3%)	10 (41.7%)		
Monthly/weekly	97 (70.3%)	41 (29.7%)		

Values are *n* (%). Chi-square tests applied. Educational level: *n* = 203 (1 missing); Income level: *n* = 201 (3 missing). * *p* < 0.05. Significant bivariate predictors: age group (*p* = 0.027), gender (*p* = 0.003), educational level (*p* = 0.006). Note on prior inconsistency: Age-stratified user counts previously summed to 113 and education-stratified to 95 because the wrong outcome variable (mobiluyg) was used during table construction. Values above are from the correct variable. Bold values indicate statistically significant associations (*p* < 0.05).

A statistically significant association was observed between gender and ChatGPT use (*p* = 0.003), with 44.6% of male participants reporting use compared to 22.3% of female participants.

ChatGPT use differed significantly across age groups (*p* = 0.027), with the highest proportion among parents aged 18–24 years (60.0%) and lower rates in older age groups.

Educational level demonstrated a significant association with ChatGPT use (*p* = 0.006). Use was reported by 20.8% of parents with high school education or below, 31.7% of undergraduate graduates, and 55.0% of postgraduate graduates.

No statistically significant association was observed between monthly household income and ChatGPT use (*p* = 0.079).

Therapy attendance frequency was not significantly associated with ChatGPT use in bivariate analysis (*p* = 0.081), though a trend was observed. Parents whose children had never attended therapy (16.7%) reported the lowest ChatGPT use, while those attending several times per year (41.7%) or monthly/weekly (29.7%) reported higher rates.

### Multivariable logistic regression analysis

**Figure 2:**
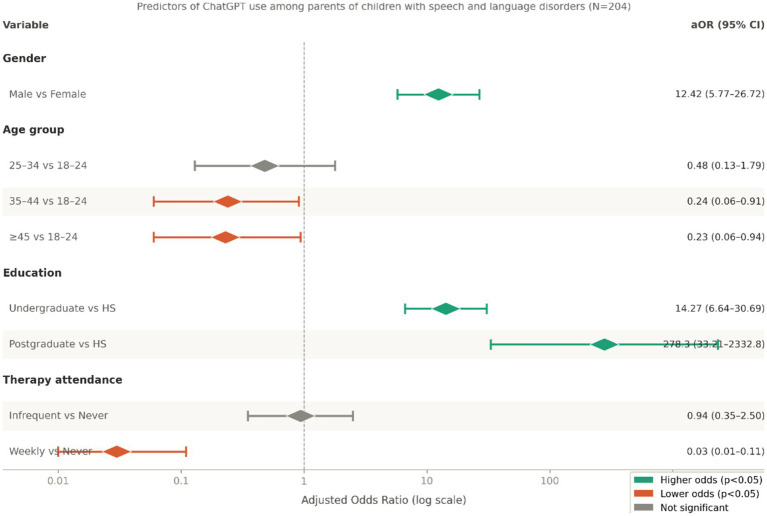
Adjusted odds ratios (aOR) with 95% confidence intervals from multivariable logistic regression analysis of predictors of ChatGPT use. Green diamonds indicate significantly higher odds (*p* < 0.05), orange diamonds indicate significantly lower odds, and blue diamonds indicate non-significant associations. HS, high school or below.

Results of the multivariable logistic regression model are shown in Table 3 and Figure 2.

**Table 3:** Multivariable logistic regression for ChatGPT use.

Variable (reference)	aOR	95% CI	P
Age group (Ref: 18–24 years)			
25–34	0.21	0.05–0.82	0.025*
35–44	0.12	0.03–0.49	0.003*
45 and above	0.10	0.02–0.48	0.004*
Gender (Ref: Female)			
Male	**3.09**	1.36–7.02	0.007*
Educational level (Ref: High school or below)			
Undergraduate	1.55	0.70–3.42	0.277
Postgraduate	**5.20**	1.56–17.27	0.007*
Income level (Ref: 0–22,000 TL)			
22,000–44,000 TL	3.38	1.10–10.33	0.033*
Above 44,000 TL	1.63	0.49–5.38	0.423
SLT attendance (Ref: Never attended)			
Several times a year	**4.01**	1.08–14.92	0.038*
Monthly/weekly	2.75	1.01–7.48	0.047*

Dependent variable: ChatGPT use (0 = No/Not aware, 1 = Yes) | *n* = 200 (complete cases). Model fit: Pseudo-R2 = 0.158 | AIC = 224.8. aOR, adjusted odds ratio; CI, confidence interval. Reference categories in parentheses. Income was retained in the model as pre-specified in the Methods section. * *p* < 0.05. Significant predictors: all age groups vs 18–24 (younger parents more likely to use ChatGPT), male gender (aOR = 3.09), postgraduate education (aOR = 5.20), income 22-44 k TL (aOR = 3.38), and attending SLT several times/year or more. Bold values indicate statistically significant associations (*p* < 0.05).

After adjustment for all included variables:

Male participants had significantly higher odds of ChatGPT use compared to females (aOR = 3.09; 95% CI: 1.36–7.02; *p* = 0.007).

Parents aged 25–34 (aOR = 0.21; 95% CI: 0.05–0.82; *p* = 0.025), 35–44 (aOR = 0.12; 95% CI: 0.03–0.49; *p* = 0.003), and ≥45 years (aOR = 0.10; 95% CI: 0.02–0.48; *p* = 0.004) all had significantly lower odds of use compared to those aged 18–24.

Undergraduate education did not reach statistical significance (aOR = 1.55; 95% CI: 0.70–3.42; *p* = 0.277), while postgraduate education was significantly associated with increased odds of ChatGPT use (aOR = 5.20; 95% CI: 1.56–17.27; *p* = 0.007).

Attending therapy several times per year was associated with significantly higher odds of ChatGPT use compared to never attending (aOR = 4.01; 95% CI: 1.08–14.92; *p* = 0.038), as was monthly or weekly attendance (aOR = 2.75; 95% CI: 1.01–7.48; *p* = 0.047).

Attending therapy several times per year was also significantly associated with higher odds of ChatGPT use (aOR = 4.01; 95% CI: 1.08–14.92; *p* = 0.038).

The regression constant was 0.103.

### Model diagnostics

Model fit was assessed using the Hosmer–Lemeshow goodness-of-fit test, which yielded a non-significant result, indicating adequate correspondence between observed and predicted values. Multicollinearity among independent variables was evaluated using variance inflation factors (VIF); all VIF values were within acceptable limits (below 2.0), confirming the absence of problematic multicollinearity in the model. The Nagelkerke *R*^2^ indicated that the model explained a substantial proportion of the variance in ChatGPT use. Overall model classification accuracy was high, with acceptable levels of both sensitivity and specificity, supporting the predictive adequacy of the final regression model.

## Discussion

Given the cross-sectional design, the findings should be interpreted as associative rather than causal, and no conclusions regarding directionality can be established. This study examined the prevalence and determinants of ChatGPT use among parents of children with speech and language disorders (SLDs) in Türkiye. Approximately one-third of participants reported using ChatGPT for disorder-related information, indicating that generative AI tools have already penetrated informal health information environments in pediatric rehabilitation contexts. However, AI adoption exhibited marked social stratification, with higher educational attainment strongly predicting AI use. These findings align with emerging evidence that AI adoption in health contexts is influenced by broader social determinants rather than being uniformly beneficial across populations.

From a TAM perspective, the findings offer partial support for the model’s core propositions. PEOU was significantly higher among ChatGPT users than non-users (Mann–Whitney U = 6273.5, *p* < 0.001), consistent with TAM’s prediction that lower perceived effort facilitates technology adoption. Users were notably more likely to endorse practical accessibility features such as 24/7 availability and the ability to provide immediate answers, suggesting that perceived ease of interaction plays a meaningful role in generative AI adoption in this context. However, PU did not significantly differentiate users from non-users (U = 4378.5, *p* = 0.637), a finding that may reflect the binary and multi-domain operationalization of usefulness items rather than a true absence of utility perceptions among adopters. Higher educational attainment and younger age—both associated with greater ChatGPT use—may independently shape the capacity to engage with and benefit from AI-generated health content, functioning as sociodemographic antecedents to TAM constructs. Taken together, these findings suggest that while TAM provides a useful interpretive lens, generative AI adoption in health contexts may be driven more strongly by perceived accessibility than by perceived informational utility, at least as operationalized in the present study. This emphasis on perceived accessibility aligns with emerging evidence from AI-enabled health systems research, where usability, system responsiveness, and integration into everyday routines are critical determinants of user adoption and sustained engagement (21).

Educational level was the most robust predictor of ChatGPT use, with undergraduate and postgraduate parents exhibiting substantially higher odds relative to those with high school education or below. This pattern reflects broader observations in the digital health literature that educational capital and digital literacy significantly shape individuals’ engagement with digital information tools (e.g., personalized AI) and influence their capacity to navigate and interpret complex outputs. Prior research has shown that health literacy and digital competence are critical determinants of effective use of health technologies and that deficits in these domains correlate with lower engagement and poorer health outcomes (22). This finding is consistent with results from a large cross-sectional study by Ayo-Ajibola et al. (19), which surveyed 2,406 adults in the United States and found that ChatGPT users for health information were significantly younger than non-users (mean age 32.8 vs. 39.1 years; *p* < 0.001). In that sample, higher advanced degree attainment was associated with lower odds of ChatGPT use (49.9% vs. 67%, *p* < 0.001), a pattern that diverges from the present study and suggests that the relationship between education and AI use may be context-dependent, shaped by national healthcare structures, sample composition, and the specific informational demands of a pediatric rehabilitation setting. Furthermore, Yun et al. (23) found that approximately 21% of adults in an international cross-sectional web-based survey used LLM-based chatbots for health information (24)—a rate lower than the 28.4% observed here—and reported no significant association between years of education and chatbot use, further underscoring the context-sensitivity of education-related AI adoption patterns. The strong association between male gender and ChatGPT use should be interpreted with caution. Some prior studies suggest that men may exhibit higher engagement with emerging and experimental technologies, whereas women may be more likely to rely on interpersonal and established health information sources. However, evidence on gender differences in digital health information-seeking behavior remains mixed and context-dependent, underscoring the need for cautious interpretation of this finding. While this finding may indicate higher engagement with generative AI tools among male participants, it may also reflect context-specific sociocultural differences in digital engagement, access, or confidence. Gender disparities in technology adoption vary across settings and are often shaped by cultural norms. Therefore, this finding should not be generalized but interpreted within its specific context.

These findings resonate with second-level digital divide frameworks, which emphasize that disparities in AI engagement reflect not merely differences in access, but in the capacity to critically use and benefit from advanced digital tools (23).

Although household income was not significantly associated with ChatGPT use in bivariate analysis (*p* = 0.079), the multivariable model revealed that middle-income parents (22,000–44,000 TL) had significantly higher adjusted odds of ChatGPT use compared to the lowest income group (aOR = 3.38; 95% CI: 1.10–10.33; *p* = 0.033), after accounting for age, gender, education, and therapy attendance. This finding suggests that income-related patterns in AI use may be partially confounded by other sociodemographic factors, and that the relationship between income and digital health information seeking warrants further investigation.

Age differences observed in this study—where older parents had lower odds of ChatGPT use—are consistent with evidence on age-related digital health engagement patterns. Research demonstrates that older adults typically exhibit lower adoption of e-health tools and require tailored interfaces and support to bridge usability gaps. Studies employing explainable AI frameworks suggest that transparent, user-centric design can help mitigate age-related disparities in interacting with digital health interfaces (25).

The association between therapy attendance and ChatGPT use in the multivariable model revealed a nuanced pattern. Contrary to expectations based on a simple substitution hypothesis, parents who attended therapy—whether infrequently (aOR = 4.01) or regularly (aOR = 2.75)—had higher adjusted odds of ChatGPT use compared to those who had never attended therapy. This finding suggests that therapy-engaged parents may not be turning away from AI tools; rather, they may be actively using ChatGPT as a complementary source of information alongside professional services. This pattern is consistent with a supplementation dynamic rather than substitution: parents already embedded in formal care pathways may be more health-engaged overall, including in their digital information-seeking behaviors. Future studies should examine whether this positive association between therapy attendance and AI use reflects increased health literacy among engaged families, clinician encouragement of supplementary digital resources, or broader patterns of proactive information-seeking behavior in this population. In contrast, a national cross-sectional survey conducted in the United States by Ayo-Ajibola et al. (20) found that adults with a usual source of primary care (USPC) were significantly less likely to use ChatGPT for health-related information (OR = 0.56; 95% CI: 0.44–0.71; *p* < 0.001) (26), suggesting an inverse relationship between formal care engagement and AI use in that context. The divergence between that finding and the positive association observed in the present study (several times/year: aOR = 4.01; monthly/weekly: aOR = 2.75) may reflect differences in healthcare system structures, the pediatric rehabilitation context, or the information-seeking profiles of therapy-engaged caregivers. These contrasting findings underscore that the relationship between formal healthcare engagement and AI use is not universal, and context-specific factors—including care setting, cultural norms, and parental health engagement patterns—warrant careful consideration in future research.

From a theoretical standpoint, these results also intersect with critical perspectives on AI and health equity. Analyses in broader healthcare contexts argue that AI systems can entrench or even exacerbate inequities due to biases in data, algorithmic decision processes, and uneven representation of diverse populations in training datasets. Without equity-centered design and governance, AI may reflect or amplify existing societal biases, leading to disparate health implications for underserved groups (27). Furthermore, advances in AI-integrated healthcare systems demonstrate that the effectiveness of such technologies depends not only on algorithmic performance but also on user interaction dynamics, system accessibility, and contextual adaptation, reinforcing the importance of aligning technological innovation with user-centered design principles (28).

While ChatGPT and similar LLMs hold considerable promise as tools for increasing informational access and supporting decision-making, current evidence underscores the need for deliberate efforts to ensure that these technologies do not inadvertently widen disparities. For instance, AI applications designed to support health literacy can be most effective when integrated into broader educational and healthcare frameworks that address underlying disparities in access and competency (16, 17).

The adjusted odds ratio associated with postgraduate education (aOR = 5.20) should be interpreted with appropriate caution given the small subgroup size (*n* = 21) and wide confidence intervals (95% CI, 1.56–17.27), which may reflect residual sparse data conditions. These values should not be interpreted as precise effect sizes but rather as indicative of a strong directional association. Future studies with larger samples are needed to obtain more stable estimates.

From a public health perspective, these findings highlight a pressing need for equity-oriented AI implementation strategies in pediatric rehabilitation and broader health services. Policymakers and technology developers should prioritize digital health literacy programs and community co-design of AI tools. Clinicians, in turn, should receive guidance on how to integrate AI-generated information responsibly into care planning discussions. The goal should not merely be technological diffusion but ensuring that generative AI enhances health outcomes equitably rather than reinforcing existing disparities. At the clinical level, speech and language therapists and rehabilitation teams are well positioned to play a proactive role in guiding families’ use of AI tools. Clinicians could routinely enquire about patients’ AI use during consultations, provide guidance on evaluating the reliability of AI-generated health information, and signpost families to validated digital resources. Integrating brief AI literacy discussions into existing parent education programs within therapy settings could represent a low-cost, scalable approach to narrowing informational disparities without requiring additional infrastructure. In Türkiye, where access to speech and language therapy services may vary substantially between urban and rural regions, integrating AI literacy into existing public health and rehabilitation services may represent a feasible and scalable strategy.

### Strengths and limitations

#### Limitations

This study has several limitations that should be considered when interpreting the findings.

First, the cross-sectional design precludes causal inference. While associations between educational level, therapy attendance, and ChatGPT use were observed, temporal or causal pathways cannot be established. Future longitudinal research is warranted to examine how AI use evolves over time in relation to healthcare engagement and outcomes.

Second, data were collected via an online survey, which may introduce selection bias. Participants with higher levels of digital engagement or greater comfort with online communication may have been more likely to participate. This may have led to an overestimation of ChatGPT use and limits the generalizability of the findings to less digitally active parents of children with SLDs. Although online methods are common in digital health research and appropriate for assessing internet-based behaviors, they may underrepresent less digitally engaged populations. Importantly, the observed strength of associations—particularly those related to education—may be partially inflated due to this selection mechanism, as digitally engaged individuals are both more likely to participate and more likely to use AI tools. Therefore, effect sizes should be interpreted as potentially overestimating population-level associations. The sample was relatively highly educated compared to national distributions in Türkiye, which may limit external validity. This overrepresentation of higher-educated participants may also contribute to the relatively high prevalence of ChatGPT use observed in this study.

Third, the operationalization of ChatGPT use relied on self-report, which may be subject to recall or social desirability bias. Participants may have interpreted the survey question about ChatGPT use differently depending on their familiarity with AI or terminology, although the instrument was piloted to enhance clarity. Moreover, the binary measurement approach does not differentiate between occasional, exploratory use and frequent, reliance-based use, which may represent qualitatively different behaviors. Future studies should distinguish between intensity and purpose of AI use to better capture its role in health information seeking. The inclusion of heterogeneous diagnostic categories (e.g., hearing loss, developmental language disorder, articulation disorder, and stuttering) represents a potential source of confounding, as information needs, parental concerns, and patterns of healthcare engagement may differ substantially across conditions. Due to sample size limitations, diagnosis-specific analyses or stratified regression models were not conducted. Future research should investigate whether diagnosis type moderates the relationship between sociodemographic factors and AI use.

Fourth, in the multivariable analysis, some categories (e.g., postgraduate education) had small cell sizes, which may have contributed to wide confidence intervals in the logistic regression model. Although the adjusted odds ratio for postgraduate education (aOR = 5.20) is more moderate than in prior model iterations, caution is still warranted given the small subgroup size. Similar challenges have been noted in other digital health adoption studies with disproportionate subgroup sizes (15).

Additionally, potentially important confounders such as digital literacy, prior technology use, and access to healthcare services were not directly measured in this study, which may have influenced the observed associations.

Finally, this study was conducted in Türkiye, a middle-income country with distinct healthcare access patterns and digital engagement norms. While this context enriches understanding of AI use outside high-income settings, findings may not be fully generalizable to populations with different healthcare systems, internet penetration rates, or cultural attitudes toward AI.

#### Strengths

Despite these limitations, the study has notable strengths.

To our knowledge, this is one of the first empirical investigations to examine parental use of generative AI tools for pediatric rehabilitation information in a sizeable sample. By integrating constructs from the Technology Acceptance Model (TAM) with sociodemographic and clinical variables, the study provides a multidimensional analysis of AI adoption.

The inclusion of therapy attendance frequency as a key variable allows for novel insights into how formal healthcare engagement relates to informal AI use. This represents an important contribution to both pediatric rehabilitation and digital health equity research, where the intersection between service access and information seeking remains understudied.

Additionally, the use of multivariable logistic regression with multiple covariates improves internal validity and helps isolate independent associations. While extreme ratio estimates warrant careful interpretation, the overall modeling approach aligns with best practices in observational epidemiology (e.g., Hosmer & Lemeshow methodology).

Lastly, the study addresses an urgent societal issue—the integration of generative AI into health information ecosystems—and situates its findings within broader digital health parity frameworks. This relevance enhances the translational value of the work for policymakers and clinicians alike.

## Conclusion

This cross-sectional study demonstrates that ChatGPT use for SLD-related health information is relatively prevalent among parents in Türkiye, with approximately one in three parents (28.4%) reporting use. However, adoption was far from uniform. Postgraduate-educated parents were significantly more likely to use ChatGPT than those with high school education or below (aOR = 5.20), while undergraduate education did not independently predict use (aOR = 1.55; *p* = 0.277). Male parents were approximately three times more likely than female parents to report use (aOR = 3.09). Notably, both infrequent (aOR = 4.01) and regular therapy attendance (aOR = 2.75) were associated with higher odds of ChatGPT use compared to never attending therapy, suggesting that parents who are engaged with formal services may also be more actively seeking supplementary information through digital tools. These findings indicate that generative AI tools, despite their potential as accessible information sources, are currently reaching those who are already more educationally and digitally privileged, while parents with lower educational attainment remain comparatively disengaged.

Generative AI has the potential to expand access to information and support family engagement in pediatric rehabilitation. However, without targeted efforts to address digital literacy and structural determinants of health, these technologies may inadvertently reinforce existing disparities in access to reliable health information. Equity-oriented strategies—such as clinician-guided AI literacy programs, integration of AI tools into formal care pathways, and community-based digital education—are needed to ensure that these benefits are distributed equitably.

Future research should examine longitudinal trends in AI adoption, evaluate the impact of AI-generated information on health outcomes, and identify effective strategies to reduce digital inequalities in healthcare settings.

## Data Availability

The raw data supporting the conclusions of this article will be made available by the authors, without undue reservation.
