# Supplementary material for: Generative AI use and social disparities in pediatric rehabilitation: a cross-sectional study of ChatGPT use among parents of children with speech and language disorders in Türkiye
Source: Front Public Health. 2026 Jun 8;14:1817290. doi: 10.3389/fpubh.2026.1817290 (PMC13283982; doi:10.3389/fpubh.2026.1817290)
Supplement: Supplementary file 1 [file Supplementary_file_1.PDF]

Generative AI Health Information Seeking and Social Inequalities in Pediatric Rehabilitation: ChatGPT Use Among Parents of Children with Speech and Language Disorders

Dear Participant,

This study is conducted by **Assist. Prof. Agit Şimşek**, Faculty Member at the Department of Speech and Language Therapy, İnönü University, and **Audiologist Zehra Güneş**. Participation is entirely voluntary, and your responses will be evaluated solely for scientific research purposes in accordance with the principles of confidentiality and anonymity. Completing the questionnaire will take only a few minutes.

Thank you in advance for your valuable contribution.

* Indicates a required question

Age * (*Please select only one option.)*

°18–24 °25–34 °35–44 °45–54 °55 and above

Gender * (*Please select only one option.)*

°Female °Male

Income Level (*Please select only one option.)*

°0–22,000 TL °22,000–44,000 TL °Above 44,000 TL

Educational Level (*Please select only one option.)*

°Primary school °Secondary school °High school °University °Postgraduate

Which region of Türkiye do you live in? (*Please select only one option.)*

°Marmara Region °Aegean Region °Central Anatolia Region °Black Sea Region

°Mediterranean Region °Southeastern Anatolia Region °Eastern Anatolia Region

Number of children (*Please select only one option.)*

°1 °2 °3 °4 or more

What is your child's age group? * (*Please select only one option.)*

°0–2 °3–5 °6–8 °9–12 °13–18

How often does your child attend speech and language therapy? * (*Please select only one option.)*

°Never attended °Once a year °Several times a year °Monthly °Weekly

What are the most common problems you encounter regarding your child's language and speech development? * *(You may select more than one option.) Please check all that apply.*

- Stuttering
- Delayed speech
- Articulation (speech sound) disorders
- Speech/language disorders related to hearing loss
- Difficulties in language comprehension
- Voice disorders (hoarseness, strained voice, etc.)
- Difficulties in social communication
- Rapid/disordered speech (pronunciation problems)
- Language and speech problems related to trauma or developmental disorders
- Other
- I have not experienced any problems

Which source do you most frequently use to obtain information about your child's language and speech development? * *(You may select more than one option.) Please check all that apply.*

- Speech and Language Therapist / Audiologist
- Internet (websites, forums)
- Mobile applications
- Family, friends, neighbors
- Books or scientific publications
- Other

Have you ever heard of the term “artificial intelligence”? *

Please select only one option.

°Yes °No

Do you use artificial intelligence applications such as ChatGPT? *

Please select only one option.

°Yes °No °I am not aware of such an application

How often do you use ChatGPT? *

Please select only one option.

°Daily °Weekly °Monthly °Less frequently °I do not use it

In which areas do you think artificial intelligence is used? **(You may select more than one option.) Please check all that apply.*

- General information search
- Education and learning
- Entertainment
- Health and therapy services
- Financial services
- Law and regulations
- Software and technology
- Other

Have you used ChatGPT to obtain information about your child’s language and speech development? *

Please select only one option.

°Yes °No °I am not aware of such an application

What types of questions did you ask ChatGPT regarding your child's language or speech disorder? * *(You may select more than one option.) Please check all that apply.*

- Information about delayed speech
- Speech exercises that can be done at home
- Ways to cope with stuttering
- Articulation/speech sound exercises
- Suggestions to support language comprehension skills
- Autism and communication
- Speech therapy methods
- Preparation for therapy or home-based support
- Other
- I did not ask any questions

What advantages do you think ChatGPT may offer in this field? * *(You may select more than one option.) Please check all that apply.*

- Early awareness and guidance
- Quick access to information
- Providing supportive activities for home practice
- Offering personalized suggestions
- Access to information 24/7
- Being a low-cost resource
- Providing practical answers to non-complex questions
- Other
- I have no opinion

What disadvantages do you think ChatGPT may have in this field? * *(You may select more than one option.) Please check all that apply.*

- Risk of providing incorrect or incomplete information
- Inability to replace a speech and language therapist
- Data privacy and security concerns
- Risk of not adequately understanding the child's individual condition
- Inability to function without internet access

- Other
- I have no opinion

Do you consult a professional (speech and language therapist, audiologist) before applying the information you obtain from ChatGPT? * *Please select only one option.*

- Yes, I always consult
- Sometimes I consult
- No, I do not consult
- I do not apply ChatGPT's suggestions
- I do not use ChatGPT

How do you evaluate the accuracy of the information provided by ChatGPT? * *Please select only one option.*

Scale: 1–10
1 = Not reliable at all 10 = Completely reliable

Do you verify the accuracy of the information you obtain from ChatGPT using another source? * *Please select only one option.*

°Yes, I always verify °No, I apply it directly °Sometimes I verify °I do not use it

Were you satisfied with ChatGPT's responses regarding language and speech development? * *Please select only one option.*

°Yes °No °Undecided °I did not ask

Do you think artificial intelligence tools such as ChatGPT should be used as a supportive tool in the field of speech and language therapy? * *Please select only one option.*

°Yes °No °Undecided
